# On-site Gram staining that increases a post-test probability of an ominous infection: a case of necrotizing fasciitis caused by *Vibrio vulnificus*: a case report

**DOI:** 10.1186/s13256-022-03731-x

**Published:** 2023-01-10

**Authors:** Mikinori Kannae, Yusuke Oka, Yohei Hamada, Toshiharu Urakami, Yosuke Aoki

**Affiliations:** 1 Division of Infectious Disease and Hospital Epidemiology, Saga University Hospital, Saga, Japan; 2 Department of International Medicine, Faculty of Medicine, Saga University, Saga, Japan

**Keywords:** *Vibrio vulnificus*, Necrotizing soft tissue infection, Pre-test probability, Gram stain, Case report

## Abstract

**Background:**

Gram staining is a classic but standard and essential procedure for the prompt selection of appropriate antibiotics in an emergency setting. Even in the era of sophisticated medicine with technically developed machinery, it is not uncommon that a classic procedure such as Gram staining is the most efficient for assisting physicians in making therapeutic decisions in a timely fashion.

**Case presentation:**

A 65-year-old Asian man with alcoholic cirrhosis complicated by esophageal varices was brought to the emergency division of Saga Medical School Hospital in early August, complaining of severe pain, redness, swelling, and purpura of the lower extremities. On physical examination he appeared in a critically ill condition suggestive of deep-seated soft tissue infection, raising a pre-test probability of streptococci, staphylococci, *Vibrio* sp., or *Aeromonas* sp. as a causative pathogen. A characteristic of his residency in an estuarine area is that raw seafood ingestion, as documented in this patient prior to the current admission, predisposes those who have a chronic liver disease to a life-threatening *Vibrio vulnificus* infection.

Given the pathognomonic clinical features suggestive of necrotizing fasciitis, our immediate attempt was to narrow down the differential list of candidate pathogens by obtaining clinical specimens for microbiological investigation, thus inquiring about the post-test probability of the causative pathogen. The Gram stain of the small amount of discharge from the test incision of the affected lesion detected Gram-negative rods morphologically compatible with *V. vulnificus*. After two sets of blood culture, intravenous meropenem and minocycline were immediately administered before the patient underwent emergency surgical debridement. The next day, both blood culture and wound culture retrieved Gram-negative rods, which were subsequently identified as *V. vulnificus* by mass spectrometry, matrix-assisted laser desorption/ionization. The antibiotics were switched to intravenous ceftriaxone and minocycline.

**Conclusion:**

The pre-test probability of *V. vulnificus* infection was further validated by on-site Gram staining in the emergency division. This case report highlights the significance of a classic procedure.

## Background

Necrotizing soft tissue infection caused by *Vibrio vulnificus* is a disease with a high mortality rate [1]. We experienced a case of *V. vulnificus* soft tissue infection presenting with septic shock. On-site Gram staining of the wound discharge right after the patient’s arrival resulted in the successful management of the severe community-acquired sepsis and was limb saving. We present a Gram staining-based treatment strategy for *V. vulnificus* necrotizing soft tissue infection.

## Case presentation

A week after seasonal heavy rainfall in early August, a 65-year-old Asian man was brought to the emergency division of Saga Medical School Hospital complaining of severe pain, redness, swelling, and purpura of the lower extremities. One day before the onset of symptoms, the patient had eaten sushi purchased at a grocery store. The patient’s past medical history was remarkable for alcoholic cirrhosis complicated by esophageal varices, chronic heart failure with atrial fibrillation, and gout. He usually received oral esomeprazole, febuxostat, furosemide, spironolactone, apixaban, brotizolam, L-isoleucine, L-leucine, L-valine, sodium ferrous citrate, and loxoprofen. He is a carpenter and reported that he had drunk 2.5 cups of sake daily. He had no specific family history. He lived alone but his sister lived close to his house.

**Fig. 1:**
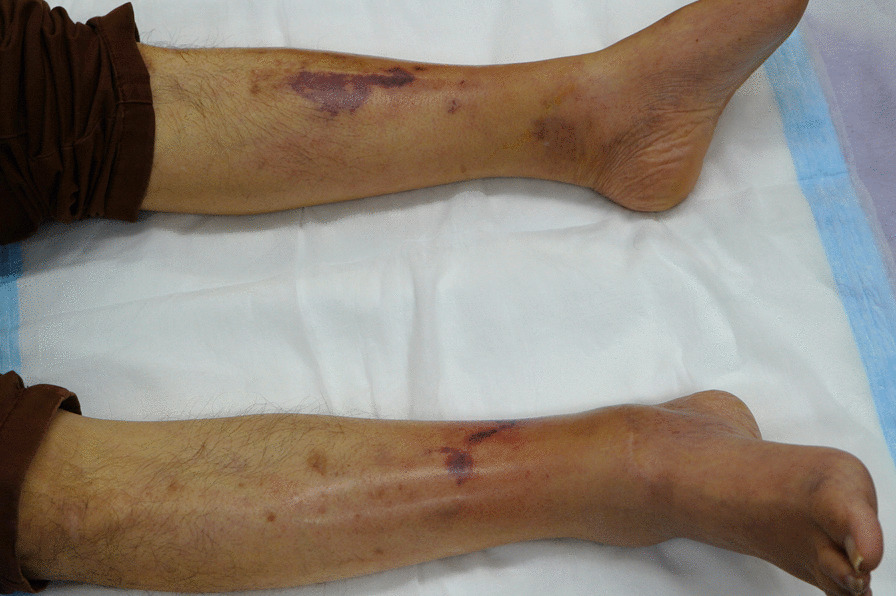
Bilateral lower legs on admission. Bilateral lower legs showed redness, swelling, and purpura accompanied by severe pain on light touch

**Fig. 2:**
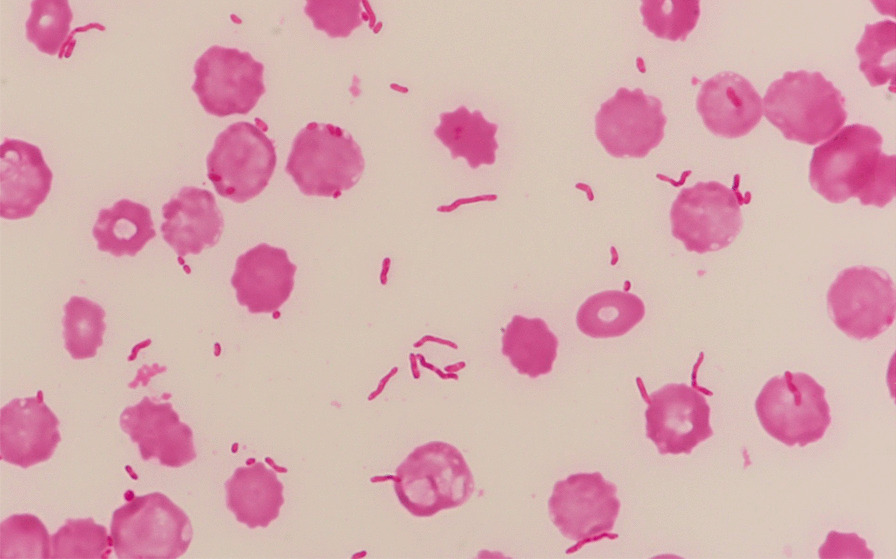
Gram stain of positive blood culture. Numerous thick and curved, banana-shaped Gram-negative rods were observed

**Fig. 3:**
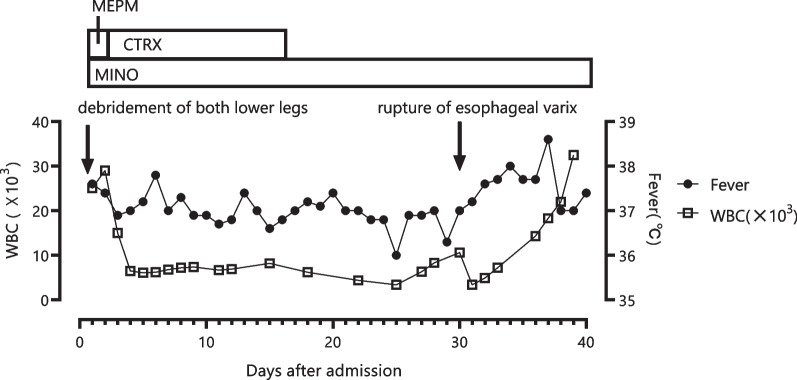
Postadmission course. Debridement of both lower extremities was performed on the day of admission. On the 30th day, esophageal varices ruptured and he died on the 40th day. *MEPM* meropenem, *CTRX* ceftriaxone, *MINO* minocycline, *WBC* white blood cells count

On arrival, he was oriented [Glasgow Coma Scale (GCS 15)], heart rate was 126 beats per minute (bpm), blood pressure 82/53 mmHg, temperature 39.5 ℃, respiratory rate 22/min, and SpO_2_ 99% (O_2_ 2 L/min). His ocular conjunctival was icteric, cervical lymph nodes were not palpated. The heart and the lungs were clear to auscultation. The abdomen was slightly distended, but soft without tenderness. Bilateral lower legs showed redness, swelling, and purpura accompanied by severe pain on light touch, a constellation of clinical features together with underlying comorbidities suggestive of necrotizing soft tissue infection (Fig. 1). His pupils were 3 mm/3 mm, the light reflex was brisk. He did not have quadriplegia. His white blood cell (WBC) count was 25,100/μL and C-reactive protein (CRP) 12.5 mg/dL. Thrombocytopenia and liver dysfunction were compatible with cirrhosis, and azotemia was noted (Table 1). The sequential organ failure score (SOFA) [2] was 9 and the laboratory risk indicator for necrotizing fasciitis [3, 4] was 8, strongly suggesting the patient was in septic shock due to necrotizing fasciitis. The small amount of discharge obtained by test incision of the affected thigh was subjected to on-site Gram-staining, in which numerous thick and curved, banana-shaped Gram-negative rods were observed (Fig. 2). Because of this characteristic microbiological morphology and the regional preponderance of Ariake Sea for *V. vulnificus*-contaminated seafoods including shellfish exclusively associated with a rainy season, a working diagnosis of *V. vulnificus* necrotizing fasciitis was considered probable. After two sets of blood cultures from both arms, the patient was put on intravenous meropenem at 3 g/day intravenously, and minocycline at 200 mg/day intravenously within 15 minutes after arrival, after which he underwent emergency surgical debridement. The blood and wound cultures were immediately transported to the laboratory and subject to both aerobic and anaerobic cultures. Because there were no fungi in the Gram stain, fungi culture was not performed. The computed tomography (CT) scan showed that fatty plaques were opacified and soft tissue density was elevated in the anterior to the medial aspect of the bilateral lower legs, suggesting fasciitis. The next day, both blood cultures (aerobic and anaerobic) and wound cultures showed Gram-negative rods and were identified as *V. vulnificus* by mass spectrometry, matrix-assisted laser desorption/ionization (MALDI TOF–MS) [5]. The antibiotics were switched to intravenous ceftriaxone at 2 g/day intravenously and minocycline at 200 mg/day intravenously (Fig. 3).

**Table 1:** Test results

Blood test results at admission					
WBC	25,100/μL	AST	88 U/L	ALP	82 U/L
Neut	94.9%	ALT	63 U/L	γ-GTP	117 U/L
Ly	1.8%	LDH	189 U/L	T. Bill	2.5 U/L
Hb	10.3 g/dL	TP	6.8 g/dL	D. Bill	0.7 U/L
Hct	35.3%	Albumin	2.8 g/dL		
Plt	64 × 10^3^/mL	BUN	41.3 mg/dL		
		Creatinine	2.35 mg/dL	pH	7.359
PT, %	35.2%	Na	129 mEq/L	PaO_2_	121 mmHg
APTT	62.1 seconds	K	4.5 mEq/L	PaCO_2_	37.9 mmHg
Fibrinogen	361.8 mg/dL	Cl	96 mEq/L	HCO_3_^−^	20.8 mmol/L
FDP	17.8 μg/mL	CK	102 U/L	BE	-3.7 mmol/L
D-dimer	8.71 μg/mL	CRP	12.5 mg/dL	Lactate	2.8 mmol/L
TAT	2.6 ng/mL				

*Hb* hemoglobin, *Hct* hematocrit, *Plt* platelet count, *PT* prothrombin time, *APTT* activated partial thromboplastin time, *FDP* fibrin/fibrinogen degradation products, *ATIII* antithrombin III, *TAT* thrombin antithrombin III complex, *AST* aspartate aminotransferase, *ALT* alanine aminotransferase, *LDH* lactate dehydrogenase, *TP* total protein, *BUN* blood urea nitrogen, *Na* sodium, *K* potassium, *Cl* chloride, *CK* creatine kinase, *CRP* C-reactive protein, *ALP* alkaline phosphatase, *γ-GTP* γ-glutamyl transpeptidase, *T. Bill* total bilirubin, *D. Bill* direct bilirubin, *PaO*_*2*_ partial pressure of arterial oxygen, *PaCO*_*2*_ partial pressure of arterial carbon dioxide, *BE* base excess

The patient’s postsurgical course was uneventful being put on oral minocycline at 200 mg/day alone until day 30 when rupture of esophageal varices developed resulting in death 10 days later due to decompensated liver failure. Because his family did not wish to, an autopsy was not performed .

## Discussion

We reported a case in which *V. vulnificus* was instantly suspected by Gram staining, and definitive treatment with meropenem plus minocycline, could be started within 15 minutes after the patient’s arrival on the first day of admission. Gram staining is said to be important [6], but there are few studies describing practical strategies. *V. vulnificus* is a Gram-negative bacterium that causes fatal bacteremia, typically complicated by necrotizing soft tissue infections [1]. Patients with chronic liver disease or hemochromatosis are at high risk for severe disease [7]. The presence of chronic medical illness and the use of steroids may worsen the prognosis of necrotizing fasciitis [8]. The natural habitat of *V. vulnificus* is brackish water and it proliferates with constant salinity and water temperature: *V. vulnificus* grows in estuaries where salinity has optimally decreased due to flooding [9], and it has been suggested that non-cholera *Vibrio* infections may increase in higher latitudes due to recent increases in the sea water temperatures [10]. The patient developed deep-seated skin/soft tissue infection in his lower legs the day after eating raw fish caught near the mouth of the river, several days after a heavy rain and flood disaster.

Given these clinical and regional epidemiological features, including temporal weather conditions of heavy rain, pointing to *V. vulnificus* necrotizing fasciitis serving as a high pre-test probability of this ominous infection, the on-site Gram-staining showing homogeneous Gram-negative rods significantly increased a post-test probability of this life-threatening infection.

There have been many previous reports that have recommended appropriate antibiotic regimens for *V. vulnificus* infection, including minocycline and ceftriaxone, or fluoroquinolones [11]. The importance of appropriate antimicrobial selection from the time of initial therapy has been emphasized in the Surviving Sepsis Campaign [12].

Rather than discussing target antimicrobial therapy following the identification of bacteria, trying to predict the most probable causative organism based on the theoretical assumption making use of fundamental clinical reasoning such as pre- or pro-test probability is not only fast and frugal, but crucially important for timely initiation of quasi-target therapy.

Although the clinical outcome of this case was poor, this does not contradict the usefulness of on-site Gram-staining in semi-identifying micro-organisms, which should continually be encouraged among emergency care physicians. This strategy will also contribute to the judicious use of antibiotics in the era of antimicrobial resistance.

## Conclusion

Necrotizing fasciitis caused by *V. vulnificus* is fatal and requires early appropriate antimicrobial therapy. Estimation of the causative organism by Gram staining is of vital importance in the treatment strategy for this life-threatening disease.

## Data Availability

The datasets used and/or analyzed during the current study are available from the corresponding author upon reasonable request.
